# 

**Published:** 1915-05-08

**Authors:** 


### CONTENTS.

PAGE

**Some Criticisms of Medical Fees** ... ... 119

**The Army, Hospital Governors and Resident Staffs** ... ... ... ... ... ... ... 120

**Hospital and Institutional News** ... ... ... 121

Mishap to a Hospital Ship—Army Chaplain as *X*-Ray Operator—Shortage of Residents at Bradford—The German Gas—A Conditional Bequest—War Pressure at Croydon General Hospital—New Designs in Respirators—Schools Used as Hospitals—Why is the Guinea Subscriber in London Asleep?—The Annual Cost of the London Hospitals—A Competition in Medical Salaries—A Suggestive Radium Week—The Medical Officer and the Workhouse Master—L. G. B. Action Called For—The Registrar-General and Mortality Statistics—The London Hospital at the Royal Academy—A Doctor's Fear of Premature Cremation—Return from the Naval Service—Metropolitan Asylums Board and the Provision of Sanatoria—Incompetent Locum Tenens—The Red Cross and the Comity of Nations—The First Poor-Law Convalescent Home—Poor-Law Medical Officers and Attendance on the Wounded—A Competition in Human Skins—The German Hospital Ship Case—This Week's Drug Market—To Our Readers.

**Radiology and Electro-Therapeutics in War-Time** ... ... ... ... ... ... ... ... 127

Lessons from the Military Hospitals.

**Painless Childbirth in Twilight Sleep** ... 128

**Modern Methods Series** ... ... ... ... 129

Lateral Spinal Curvature—Urethroplasty.

**The Administration of the Lunacy Acts** ... 130

Are Further Safeguards Needed?

**Enterprise in American Hospital Management** ... ... ... ... ... ... ... 131

The Hutchinson Museum and its Lessons.

**Research and Remedies** ... ... ... ... 132

Isolation Hospitals and Cerebro-spinal Meningitis.

Puerperal Septicæmia.

Sphygmometer Records in Relation to Life Insurance.

[*See over.*]

**We can supply immediately from stock for Emergency Hospitals**

| | |
|---|---|
| Bedsteads | Waterproof Sheetings |
| Mattresses | Towellings |
| Blankets | Flannels |
| Linen & Cotton Sheetings | Calicoes, &c., &c., &c. |

**D. STEWART & CO.**

16 Staining Lane, Gresham Street, London, E.C.

**CONTRACTORS TO HOSPITALS, SANATORIA, &c.**

Telephone: BANK 521. Telegraphic Address: "CENTRALIZE, CENT, LONDON."

### CONTENTS—(*continued*).

| | PAGE |
|---|---|
| **Hospitals and the Spirit Tax** ... ... ... | 133 |
| The Chancellor's Difficulties and a Way Out. | |
| **Albert Thomas, our First Hospital Warrior** ... | 134 |
| His Memorial, his Work and his Friends. | |
| **Round the World** ... ... ... ... ... | 135 |
| Fellow-Workers and the Roll of Honour. | |
| **The Hospitals and Dispensaries of Bengal** ... | 136 |
| **Hospital Architecture and Construction** ... | 137 |
| Derbyshire Children's Hospital. | |
| **Public Pharmacists' Session Ends** ... ... | 137 |
| **The Department of Modern Nursing** ... ... | 138 |
| XII.—The Royal Devon and Exeter Hospital. | |
| **An X-Ray Installation for a War Hospital** ... | vi |
| **The Royal College of Physicians** ... ... ... | vi |
| **Gifts and Bequests** ... ... ... ... ... | vi |
| **Medical Appointments** ... ... ... ... | vi |
| **The Book Market** ... ... ... ... ... | vi |
| **A Doctor's Bequests** ... ... ... ... ... | vi |
| **The Institutional Worker** ... ... (Suppl.) | 1 |
| VI.—The Chief Clerk. | |
| Question for May. | |
| Enquiries and Answers. | |
| Employment and Training. | |
| The Housekeeper's Department. | |
| The Sick and in Need. | |

